# Intact DNA purified from flow-sorted nuclei unlocks the potential of next-generation genome mapping and assembly in *Solanum* species

**DOI:** 10.1016/j.mex.2018.03.009

**Published:** 2018-04-10

**Authors:** Paola Gaiero, Hana Šimková, Jan Vrána, Federico F. Santiñaque, Beatriz López-Carro, Gustavo A. Folle, José van de Belt, Sander A. Peters, Jaroslav Doležel, Hans de Jong

**Affiliations:** a Faculty of Agronomy, University of the Republic, Montevideo, Uruguay; b Laboratory of Genetics, Wageningen University & Research, Wageningen, The Netherlands; c Centre of Plant Structural and Functional Genomics, Institute of Experimental Botany, Olomouc, Czech Republic; d Flow Cytometry and Cell Sorting Core, Instituto de Investigaciones Biológicas Clemente Estable (IIBCE), Montevideo, Uruguay; e Applied Bioinformatics, Department of Bioscience, Wageningen University & Research, Wageningen, The Netherlands

**Keywords:** Nuclei sorting and HMW DNA purification in Solanum, HMW DNA isolation, Flow sorting, BioNano genome mapping, Genome finishing

## Abstract

Next-generation genome mapping through nanochannels (Bionano optical mapping) of plant genomes brings genome assemblies to the ‘nearly-finished’ level for reliable and detailed gene annotations and assessment of structural variations. Despite the recent progress in its development, researchers face the technical challenges of obtaining sufficient high molecular weight (HMW) nuclear DNA due to cell walls which are difficult to disrupt and to the presence of cytoplasmic polyphenols and polysaccharides that co-precipitate or are covalently bound to DNA and might cause oxidation and/or affect the access of nicking enzymes to DNA, preventing downstream applications. Here we describe important improvements for obtaining HMW DNA that we tested on *Solanum* crops and wild relatives. The methods that we further elaborated and refined focus on

• Improving flexibility of using different tissues as source materials, like fast-growing root tips and young leaves from seedlings or *in vitro* plantlets.
• Obtaining nuclei suspensions through either lab homogenizers or by chopping.
• Increasing flow sorting efficiency using DAPI (4′,6-diamidino-2-phenylindole) and PI (propidium iodide) DNA stains, with different lasers (UV or 488 nm) and sorting platforms such as the FACSAria and FACSVantage flow sorters, thus making it appropriate for more laboratories working on plant genomics.

The obtained nuclei are embedded into agarose plugs for processing and isolating uncontaminated HMW DNA, which is a prerequisite for nanochannel-based next-generation optical mapping strategies.

## Graphical abstract

Nuclei sorting and High Molecular Weight (HMW) DNA isolation workflow. Nuclei suspensions are prepared by homogenization or chopping of root tips from germinated seeds or leaves from seedlings or plants, either potted or *in vitro*. Nuclei are sorted by selecting the G1-S-G2 populations through a FACS Aria or FACS Vantage flow sorter. Flow sorted nuclei are embedded in agarose plugs and HMW DNA is purified with proteinase K and RNAse A and then isolated. Pulsed-field gel electrophoresis (PFGE) is used for DNA quality control. HMW DNA is subsequently label and analyzed on the Irys platform. Optical mapping (OM) data are then processed for de novo OM and hybrid assemblies.

**Figure:**
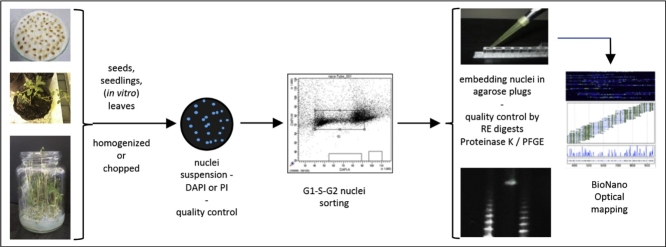

Specifications Table

**Table:** 

Subject area	*Select one of the following subject areas:*
	• *Agricultural and Biological Sciences*
More specific subject area	*DNA purification, Genomics*
Method name	*Nuclei sorting and HMW DNA purification in Solanum*
Name and reference of original method	*H. Šimková, J. Číhalíková, J. Vrána, M.A. Lysak, J. Doležel.*
	***Preparation of HMW DNA from plant nuclei and chromosomes isolated from root tips***
	*Biol. Plant., 46 (2003), pp 369–373*http://link.springer.com/article/10.1023/A:1024322001786
Resource availability	Flow sorter (FACS Aria or FACS Vantage, BD Biosciences, Santa Clara, USA)
	Polytron PT1200 homogenizer
	Low-melting point (LMP) agarose (Bio-Rad, 1613111) and plug molds (Bio-Rad, 1703713)
	Pulsed field gel electrophoresis equipment (CHEF-DR II system, Bio-Rad or BluePippin, Sage Science)

## Method details

### Plant material and preparation of nuclei suspensions (for two plugs with 500,000 nuclei each)

The whole workflow for this method is summarized in the graphical abstract. The method follows Šimková et al. with the following modifications. First of all, we introduced variations in the starting material types. For tomato (*Solanum lycopersicum*), we used root tips, for *S. commersonii* young leaves from *in vitro* plantlets and for *S. etuberosum* young leaves from plants grown in pots. Second, we tested different options to obtain nuclei suspensions, namely homogenizing with a Polytron or chopping with a razor blade. The protocol for tomato root tips is as follows:

Germinate about 200 seeds on humidified filter paper in Petri dishes for about 4 days.

Add 1.1 μL β-mercaptoethanol per 1 mL of 1.5x isolation buffer (IB [1]: 15 mM Tris, 10 mM EDTA, 130 mM KCl, 20 mM NaCl, 1 mM spermine, 1 mM spermidine and 0.1% Triton X-100, pH 9.4) just before use.

Transfer the seedlings to a 2% formaldehyde solution (from stock solution 36.5–38 % in H_2_O, SIGMA F8775) in Tris buffer (10 mM Tris, 10 mM EDTA, 100 mM NaCl, 0.1% v/v Triton X-100, pH 7.5). Incubate in a water bath at 4 ℃ for 20 min and wash three times in Tris buffer at 4 ℃ for 5 min.

Dissect 1–2 cm of the root tips on a glass Petri dish, divide the material between two 5 mL polystyrene tubes containing 1 mL ice-cold 1.5x IB with β-mercaptoethanol and keep on ice.

Homogenize samples using a Polytron PT1200 homogenizer at 15,000 rpm for 13 s (time and speed adjustable according to species).

The alternative protocol for *S. commersonii* and *S. etuberosum* young plant leaves is as follows:

Fix whole *in vitro* grown plantlets or detached leaves from potted plants in formaldehyde solution as described above.

After rinsing, place 0.5–1 g of leaves in a glass Petri dish with 1 mL of 1.5× IB buffer

Chop the tissues using a sharp razor blade until a soft homogenate is obtained. This should be formed by very small pieces of leaves in a green suspension. Continue from step 1.6.

Filter the crude homogenates through a 50 μm nylon mesh into a new polystyrene tube and a 25 μm nylon mesh (Silk & Progress, 130 T EXTRA, www.silkandprogress.cz), respectively. Alternatively, samples can be filtered through a Falcon® 40 μm cell strainer (Corning Life Sciences, Oneonta, New York, Product #352,340). Collect the filtered nuclei suspensions aliquots up to a volume of ∼4 mL.

Add DAPI to a final concentration of 2 μg mL^−1^. Check nuclei integrity and concentration under the fluorescence microscope equipped with appropriate excitation and emission filters. Nuclei should be round-shaped, not broken and at a density of 150–200 nuclei per mm^2^ (at 10× magnification).

Keep samples on ice until flow cytometric analysis and sorting.

### Nuclei flow sorting

For flow sorting, we adjusted the protocols of Šimková et al. and Vrána et al. to allow the use of either the FACS Aria or the FACS Vantage flow sorters. We introduced the following modifications:

Stain the nuclei with DAPI (final concentration: 2 μg mL^−1^)

Sort DAPI-stained nuclei using a FACSAria II SORP (BD Biosciences, San José, USA) with the following settings:

a) Solid-state laser in the UV range (355 nm, 100 mW); b) 70 μm nozzle, 70 psi; c) sorting speed: 300 events/s.

We performed data acquisition and analysis with the BD FACSDiva software (BD Bioscences, San José, CA, USA)

**Fig. 1:**
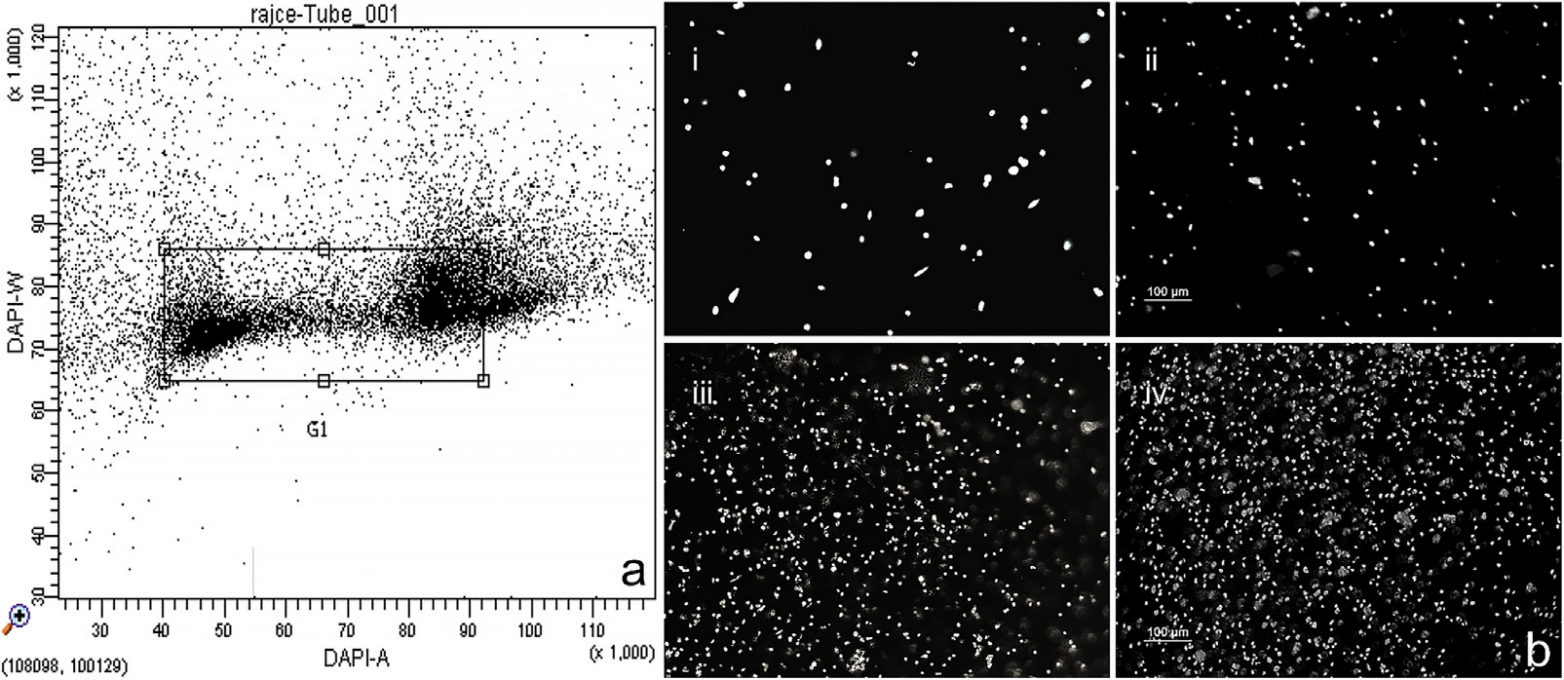
a.Dotplot of DAPI- A (x axis) vs DAPI-W (y axis), framed dot clouds are *Solanum* nuclei in G1 + S + G2 to be sorted. b. DAPI or PI stained *Solanum* nuclei from samples throughout the workflow under epifluorescence microscope: i. nuclei suspension after flow sorting, ii. pelleted nuclei after centrifugation at 500*g* for 30 min, iii. pellet mixed with LMP agarose, iv. slice from a plug with nuclei already embedded. Scale bars represent 100 μm and apply to all panels in the figure.

Select the G1, S and G2 nuclei for sorting using DAPI-A vs DAPI-W dot plots (Fig. 1a).

In order to use the FACS Vantage (BD Biosciences, SanJosé, USA), the following protocol for staining with PI was used:

Stain the nuclei with Propidium Iodide (PI, Sigma Aldrich, P4170) (final concentration: 50 μg mL^−1^) in the dark for at least 10 min prior to the flow cytometry measurements.

Sort PI-stained nuclei using a FACSVantage cytometer operated with these settings:

a) Argon-ion Innova 304 Laser (Coherent, USA) (488 nm, 100 mW); b) 70 μm nozzle; c) sorting speed 500 events/s in counter sort mode.

We performed data acquisition and analysis with the CellQuest software (BD Bioscences, San José, USA)

Select PI-stained G1, S and G2 nuclei populations for sorting using PI-A vs PI-W dot plots, avoiding the inclusion of debris (Fig. 1a).

In both sorting platforms, use a 50 mM NaCl solution in MQ (milli-Q, Millipore Corporation) water as sheath fluid. Collection tubes for the sorted nuclei contain 400–500 μL of ice-cold 1.5× IB. This volume should be equal to the volume that comes with the sorted fraction and depends on the sorted-droplet volume and number of sorted nuclei. Keep the samples as well as collection tubes at 4 ℃ during sorting using a precision refrigeration unit (±0.2 ℃) connected to the flow sorter.

### Agarose plugs preparation and quality controls

Plug preparation was performed following Šimková et al. We modified the centrifugation steps (speed and time) to make them more efficient to recover *Solanum* nuclei.

Pellet nuclei (400,000–500,000 per tube) at 500 g and 4 ℃ for 30 min.

Two fluorescence microscopic checkpoints can be optionally introduced, before and after pelleting, for nuclei integrity and concentration after staining with 2 μg mL^−1^ DAPI or 50 μg mL^−1^ PI (Fig. 1b).

Discard supernatant keeping 15 μL in the tube with the pellet. Gently resuspend pelleted nuclei in the leftover supernatant.

Warm the nuclei suspension and keep at 52 ℃ for 3–5 min

Mix with 8.5 μL 2% low-melting point (LMP) agarose (Bio-Rad, 1613111) dissolved in 1x IB.

Keep the mixture at 52 ℃ for another 5 min and then slowly pour the mixture into pre-warmed plug molds (Bio-Rad, 1703713) using a wide bore pipette tip.

Solidify the plugs at 4 ℃ for 10 min

Push the plugs into a polystyrene tube with 500 μL per plug of lysis buffer C (0.5 M EDTA, 1% N-lauroyl-sarcosine (Sigma Aldrich, L5000)). Add freshly prepared proteinase K (Sigma Aldrich, P6556) to reach a final concentration of 0.3 mg/mL (30 μL of stock solution per 1 mL). For the two washes, prepare 62 μL of stock solution per 1 mL of buffer. Weigh 62 μg and dilute in 62 μL of MQ (milli-Q, Millipore Corporation) water. Before weighing, let the proteinase K adapt to room temperature to avoid condensation on the powder. Add 30 μL for the first wash and keep the rest at 4 ℃ for the second wash.

Incubate at 37 ℃ for 24 h under gentle shaking (50 rpm) in an almost horizontal position.

Change for lysis buffer B (0.5 M EDTA, 1% N-lauroyl-sarcosine (Sigma Aldrich, L5000), 0.3 mg mL^−1^ proteinase K (Sigma Aldrich, P6556, pH 8.0) and incubate plugs for another 24 h under the same conditions.

After the proteinase K treatment, rinse and store agarose plugs in ET buffer (1 mM Tris, 50 mM EDTA, pH 8.0) at 4 ℃.

**Fig. 2:**
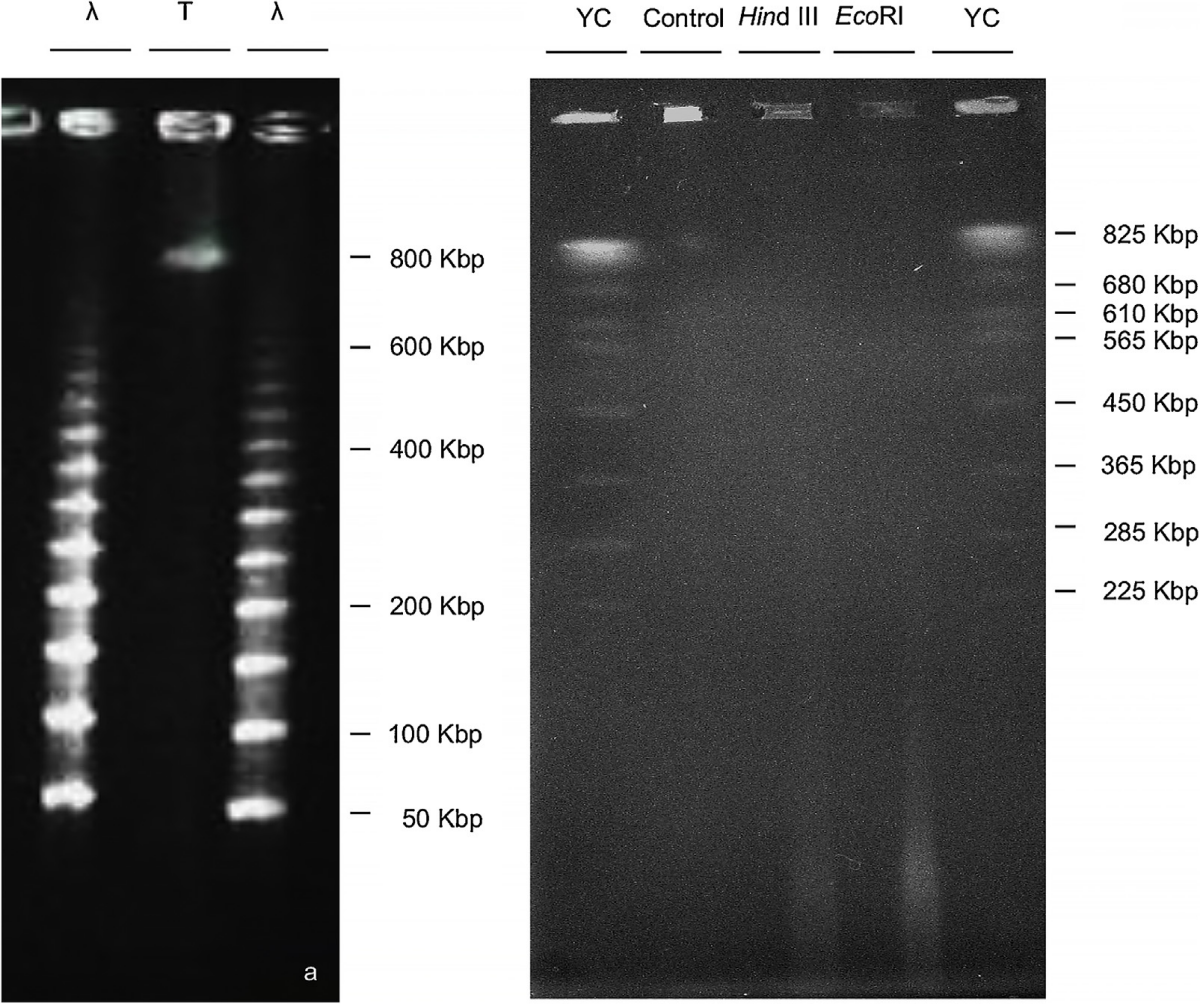
a. Quality check of *Solanum lycopersicum* HMW DNA (h) by PFGE. λ - Lambda Ladder, unit size 48.6 kb. b. PFGE of restriction enzyme digestion of *Solanum commersonii* HMW DNA for accessibility test (cropped image). YC- yeast chromosomes, Control - HMW DNA, 20 min at 37 ℃ in digestion buffer without restriction enzymes, *HindIII* and *Eco*RI, 2 U for 20 min at 37 ℃.

Check DNA quality using pulsed field gel electrophoresis (e.g. CHEF-DR II system, Bio-Rad or BluePippin, Sage Science) (Fig. 2a and b).

The plugs obtained were ready for RNAse treatment, DNA release and labeling following the standard protocol recommended by the genome mapping platform manufacturer (BioNano Genomics).

## Assessment of DNA quality

We included various quality checkpoints throughout the workflow. The integrity of nuclei was checked before and after flow sorting. Fluorescence microscopy revealed intact nuclei, with regular shape and a suitable density for isolating DNA, evenly distributed with about 1500–2000 nuclei per mm^2^ at 10x magnification (Fig. 1b, i and ii).

Following nuclei embedding in agarose plugs we checked nuclei features again (Fig. 1b, iii and iv). High density of round, regular shaped nuclei was obtained. DNA quality and size after the proteinase K treatment was checked through Pulsed-Field Gel Electrophoresis (PFGE) (Fig. 2a). DNA was protein-free (no fluorescence in the slots) and with molecule size equal to or larger than 800 Kbp. DNA accessibility for enzymes was checked by digestion with restriction enzymes (*Hind*III and *Eco*RI, 2 U in Digestion buffer (DB, 1x enzyme buffer, 1 mM DTT, 4 mM spermidine, 0.39 mg BSA) for 20 min at 37 ℃). DNA was readily accessible for restriction enzyme digestion even at low concentrations, confirming its suitability for physical mapping (Fig. 2b).

**Fig. 3:**
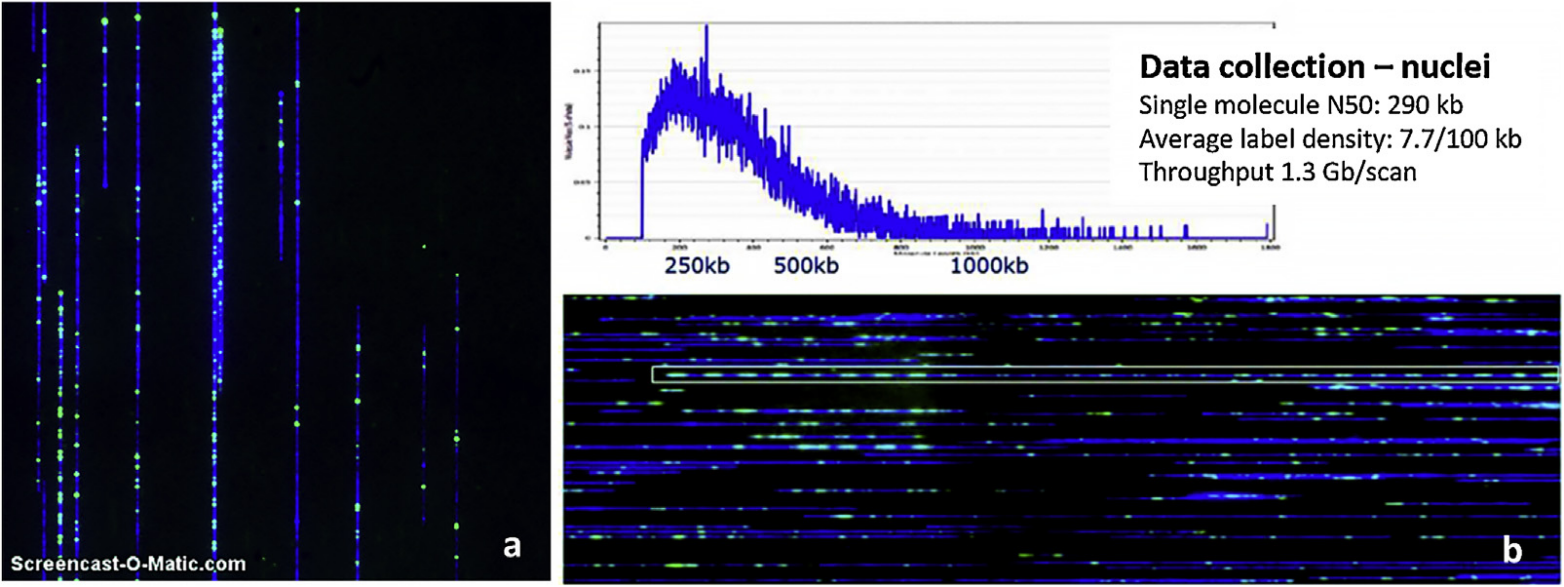
a. Snapshot of linearized labeled DNA fragments running in nanochannels for *Solanum lycopersicum.*b. Size distribution of molecules and N_50_ stat for *S. lycopersicum.* The white box highlights a tandem repeat of approximately 200 kbp.

Plugs with high quality DNA were RNAse treated and DNA was released from the plugs and labeled following the standard protocol recommended by BioNano Genomics. The labeled DNA was imaged on the Irys platform (Fig. 3a and b). Taking tomato cv Heinz 1706 as an example, we found single molecule N_50_ lengths of 290 kb and DNA quality allowing a labeling density of 7.7 sites per 100 Kbp from 12 sites per 100 Kbp predicted *in silico.* Additionally, there was no clogging of the chips, thus allowing a throughput of 1.3 Gbp per scan (size-filtered molecules >150 Kbp) (Fig. 3a and b).

## Advantages over comparable methods

Production of HMW DNA of superior quality, i.e., molecules of several hundred kilobases, has been identified as the bottleneck in nanochannel-based genome mapping technologies (BioNano Genomics). Our method yields the longest DNA molecules on average (290 kbp, compared to the 160 Kbp molecules and 7.5 sites per 100 Kbp obtained through other methods, unpublished results). Run costs become lower, as longer molecules mean that less coverage is needed when doing genome mapping. Moreover, longer molecules with proper labeling frequency imply higher N_50_ in the consensus genome map. Complex regions in the genome can be spanned and resolved better, which contributes to the contiguity of the assembly.

Contamination by cytosol inclusions in different plant cell types represents another important impediment to the use of optical mapping. Such cytoplasmic compounds, mostly phenolic compounds, polysaccharides and other secondary metabolites co-precipitate with DNA and interfere with enzymes used in DNA labeling. When nuclei are flow-sorted, the contamination with such substances is minimized, and so ultimately leads to fewer false negatives in optical mapping. Purity also reduces clogging of nanochannels, thus allowing for higher throughput since it extends chip lifetime. In terms of cytoplasmic contaminants, flow sorting yields HMW DNA of much higher purity compared to other methods, without cell debris and very low amounts of chloroplast and mitochondria contamination [2], which could represent a problem because in large leaves the amount of chloroplast DNA is often much higher than that of nuclear DNA [3].

The yield of pure, HMW DNA from the tomato cv Heinz 1706 material (genome size 950 Mbp) amounted about 1.6 × 10^6^ nuclei in four agarose miniplugs, which was enough to produce 73 Gbp of size-filtered data with single molecule N_50_ of 290 Kbp on the Irys platform. We obtained this sample of nuclei in 3 h of sorting in a FACSAria or 6 h of sorting in a FACSVantage flow cytometer. These results confirm the excellent quality of nuclear plant DNA obtained through flow sorting which is similar to the quality obtained from mammalian cell cultures. Moreover, molecule size distribution was on par with human samples (personal communication, BioNano Genomics).

## Implications for research and breeding

Flow sorted nuclei provide a good starting point for mapping and sequencing technologies where high purity and megabase-sized DNA is required. The protocol [1] that we modified was originally developed for construction of BAC libraries, but is equally suitable for optical mapping. We applied our optimized method to *Solanum* crops and wild relatives, in order to further improve the quality of genome sequencing and assembly, and for comparative structural genomics including related crops and wild relatives. We introduced relevant modifications that enhanced both efficiency and versatility of this method. The main adjustments are related to the use of different source materials (root tips or young leaves from seedlings or i*n vitro* plantlets), different methods to obtain nuclei suspensions (homogenization or chopping), two DNA-specific fluorescence dyes (DAPI and PI) with their corresponding lasers (UV and 488 nm or 514 nm) and both classic and modern sorting platforms (FACS Vantage or Aria). With these modifications, we expect that the method is also successful in different sorting platforms and laser configurations, meaning that laboratories without access to the latest flow sorting technology still can have access to next-generation mapping. One point of extra attention when following this protocol using sorting platforms is that only standard UV lasers and DAPI can be used, as the formaldehyde in the fixed nuclei interferes with PI fluorescence. However, the histograms obtained in this work were clear and well defined, and had low CVs (coefficients of variation – ratio of the standard deviation to the mean) of DNA peaks. The possibility to use either mechanical homogenization or manual chopping allows for flexibility depending on the plant species. For example, nuclei from *S. commersonii* leaves better preserve their integrity and carry less debris when obtained by chopping with a razor blade than using a Polytron whereas this kind of homogenizer is ideal for tomato root material.

In the case of elite or proprietary breeding material, seed propagation is often not possible or it is necessary to preserve the genotype to be analyzed so the only way to obtain enough material is through vegetative propagation. It would not be possible to isolate HMW DNA from this kind of materials from embryonic root tips. The modifications included in this method allow for isolation of nuclei from young leaf material, thus enabling the use of genome mapping for breeding lines that are propagated vegetatively.

In conclusion, the workflow proposed here involving the coupling of flow sorting with nanochannel-based mapping will allow this genome mapping technology to fulfill its potential in plant genomics and genomics-based breeding.

## Supplementary material *and/or* additional information

High-throughput NGS technologies have enabled the *de novo* sequencing of an increasing number of plant species. However, nearly-finished well-assembled genomes are not easy to obtain. Issues related to order and orientation of contigs and distribution of repetitive sequences remain major challenges [4]. Genome sequences from non-model species, orphan crops or even main crops with larger or more complex genomes are still far from finished. Genome studies are lacking structural comparisons, since the focus of most resequencing efforts has been on SNP variation and, at best, on microsynteny [5]. Among several developments that aim to facilitate genome assembly (such as chromatin conformation capture or Hi-C [6] or Chicago libraries by Dovetail Genomics [7]), the new next-generation genome mapping technologies (BioNano Genomics Irys) [8,9] have provided significant improvements across a broad range of organisms. They can improve assembly metrics such as N_50_ or percentage of whole genome assembled, by sizing and/or closing gaps, scaffolding, joining scaffolds, correcting assembly errors and even identifying, spanning and assembling repeated sequences. In addition, genome mapping can in its own right provide a comprehensive assay system for defining structural variation among related species or genotypes within a species [9].

The nanochannel-based genome mapping technology has been described extensively [8,9]. This technology uses nicking enzymes to create single strand DNA sequence-specific cuts that are subsequently labeled by a fluorescent nucleotide analog upon repair of the nicks by a DNA polymerase [10]. The nick-labeled DNA is stained with the intercalating dye YOYO-1, loaded onto the nanofluidic chip by an electric field, and imaged with high N.A. optics and a CCD camera. The DNA is linearized by confinement in a nanochannel array [11], resulting in uniform linearization and allowing precise and accurate measurement of the distance between nick-labels on DNA molecules comprising a signature pattern. Also, the DNA loading and imaging cycle can be repeated many times in a completely automated fashion; data can be obtained at high throughput and high resolution [12]. It builds on the earlier optical mapping technologies overcoming many of their limitations, particularly in terms of throughput, resolution and precision of distance measurements [12,13].

Despite all these advantages, nanochannel-based genome mapping has been used only recently for the assembly of DNA in higher plants such as spinach [14], subterranean clover [15], maize [16], quinoa [17] and bread wheat [18], with HMW DNA isolation in most cases as the bottleneck for its application. Previously, a related method called optical mapping [19,20] was used for whole genome analysis in crops like rice [21], maize [22] and tomato [4] and for crop relatives such as *Medicago truncatula* [23,24]. It has also been applied to validate assembly of a 2.1-Mb prolamin gene family region from the genome of *Aegilops tauschii* [12] and more recently to evaluate the quality of the whole genome hybrid assembly from this wheat progenitor [25].

Setting aside issues of genome size and complexity and computational limitations, one of the main bottlenecks for the application of nanochannel-based genome next-generation mapping to plant genomes is the requirement of high quality HMW DNA. Such DNA is easier to obtain from mammalian cells than from plant cells, because of many important differences in their composition. The rigid cell walls in plant cells demand for mechanical methods to disrupt them, which can cause shearing of the DNA. There are various contaminants in plants that are not found in mammalian cells, such as chloroplasts and a range of secondary metabolites which contaminate the DNA sample during the precipitation process [26]. Sometimes plants rich in secondary metabolites are the most interesting from the point of view of breeding, since these metabolites might be the breeding target and/or confer resistance to pests and diseases [26]. It was reported previously that nuclei and chromosomes purified by flow cytometric sorting provide quality HMW DNA even in species rich in secondary metabolites [1,27]. Finally, the higher prevalence of polyploidy in plants affects DNA yield.
